# Increased Intraoperative Vasopressor Use as Part of an Enhanced Recovery After Surgery Pathway for Pancreatectomy Does Not Increase Risk of Pancreatic Fistula

**DOI:** 10.1089/pancan.2018.0007

**Published:** 2018-06-01

**Authors:** Shachar Laks, Robert S. Isaak, Paula D. Strassle, Lyla Hance, Lavinia M. Kolarczyk, Hong Jin Kim

**Affiliations:** ^1^ Department of Surgery, East Carolina University, Greenville, North Carolina.; ^2^ Department of Anesthesiology, University of North Carolina, Chapel Hill, North Carolina.; ^3^ Department of Surgery, University of North Carolina, Chapel Hill, North Carolina.

**Keywords:** vasopressors, ERAS, pancreatectomy, pancreatic fistula, anastomotic leak, enhanced recovery after surgery

## Abstract

**Purpose:** Enhanced recovery after surgery (ERAS) pathways are increasingly implemented. Goal directed fluid therapy (GDFT) is a core component of ERAS pathways that limit excessive volume administration and is associated with increased use of intraoperative vasopressors. Vasopressor effects on anastomotic healing and pancreatic fistula are inconclusive. We hypothesized that intraoperative vasopressor use in an ERAS GDFT algorithm would not increase risk of pancreatic fistulas.

**Methods:** We reviewed all adult patients undergoing pancreatectomy at an academic institution from January 2013 to February 2016, before and after implementation of an ERAS pathway in July 2014. Retrospective chart review was performed. Log-binomial regression, weighted by stabilized inverse probability-of-treatment weights, estimated effect of ERAS and intraoperative vasopressors on fistula risk.

**Results:** One hundred thirty two patients met inclusion criteria: 74 (56.1%) in the ERAS cohort. No significant differences in overall leak risk (risk ratio [RR] 0.89, 95% confidence interval [CI] 0.38–2.09) were observed between the ERAS and pre-ERAS cohorts. Similarly, vasopressor infusions, independent of ERAS pathway, did not significantly increase the risk of anastomotic leaks (RR 1.19, 95% CI 0.52–2.72).

**Conclusions:** Increased use of vasopressor infusions as part of an ERAS pathway for pancreatic surgery is not associated with an increase in the risk of clinically significant pancreatic fistulas.

## Introduction

Enhanced recovery after surgery (ERAS) pathways have been increasingly implemented for a variety of major surgeries over the past decade and demonstrated to decrease length of stay, provide superior analgesia, reduce costs, and decrease morbidity.^1–7^ ERAS pancreatectomy pathways have decreased hospital length of stay and improved outcomes.^8–10^

A universal concept in ERAS pathways is avoidance of excessive crystalloid administration, often accomplished using a goal directed fluid therapy (GDFT) algorithm.^11–12^ GDFT algorithms evaluate hemodynamic parameters using real-time interpretation of dynamic indices such as pulse pressure or stroke volume variation. The aim of a GDFT algorithm is to maximize cardiac output by optimizing preload and contractility, which in turn ensures adequate vital organ perfusion maintaining intracellular oxygenation. Fluid administration is typically the first step in treating intraoperative hypotension. However, only preload is appropriately addressed when administering fluids. In ERAS pathways, hypotension can often be the result of low systemic vascular resistance (SVR) and should subsequently be treated using vasopressors as part of a GDFT algorithm. As such, intraoperative vasopressor infusions play an increased role in the intraoperative management of hypotension for patients managed using a GDFT as part of an ERAS pathway.

Historically, there has been concern in the surgical community about the risk vasopressors pose to anastomotic healing. Appropriate healing of gastrointestinal anastomoses requires appropriate blood supply and oxygenation.^13–15^ Vasopressor use is associated with splanchnic vasoconstriction and thus gastrointestinal hypoxia and injury.^16–18^ Some authors have demonstrated an association between perioperative vasopressor use and anastomotic leaks.^19–21^ Yet experimental animal models of intraoperative vasopressor use fail to demonstrate an effect on intestinal leak or anastomotic burst pressure.^22^ To our knowledge, there has not been any published literature addressing the relationship between pancreatic fistulas and intraoperative vasopressor use. We hypothesized that increased use of intraoperative vasopressors, as part of a GDFT algorithm for ERAS pathways for pancreatic surgery, would not correlate with an increased risk of pancreatic fistula.

## Methods

After approval from the University of North Carolina Institutional Review Board (IRB#14-0831, 5/19/2014), and in compliance with the guidelines of the responsible governmental agencies, we compared prospective data from consecutive patients undergoing pancreatic surgery under an ERAS pathway (ERAS cohort) to a retrospective cohort of patients undergoing the same pancreatic surgical procedures before the implementation of the ERAS pathway (pre-ERAS cohort). The ERAS pathway for pancreatic surgery was implemented in July of 2014 and collected data prospectively through February 2016. The pre-ERAS cohort consisted of patients from the preceding 18 months (January 2013 to June 2014). Patients with total pancreatectomies were excluded from the analysis. Patients with end-stage renal disease requiring dialysis and patients less than 18 years old were also excluded from the analysis. A single experienced pancreatic surgeon performed all procedures in both groups, to minimize variability in technique and clinical outcomes. Pancreaticojejunostomy anastomosis was created in a similar manner in all patients. This included a two-layer pancreatic duct to mucosa side-to-end anastomosis with interrupted 3-0 silk sutures to the pancreatic parenchyma to jejunal subserosal layer and 5-0 or 6-0 interrupted polydioxanone sutures for the duct to mucosa layer. Two 15Fr Blake drains were placed at time of pancreaticoduodenectomy, one anterior and one posterior to the anastomoses. Distal pancreatectomy was accomplished in a similar manner in all patients with a Covidien Endo GIA 60 mm reinforced black (extra thick) stapler load. A single 15Fr Blake drain was placed adjacent to pancreatic staple line.

### ERAS pathway

ERAS pathway for pancreatic surgery included evidence-based best practices for preoperative, intraoperative, and postoperative care and was modeled after previously published ERAS pathways for pancreatic surgery.^7–9^ Important preoperative components included the following: consumption of a carbohydrate drink before surgery, preoperative 1 L bolus of Lactated Ringers, placement of a low thoracic epidural, and administration of opioid-sparing multimodal analgesics that included acetaminophen, celecoxib, and pregabalin. Intraoperative components included the following: venous thromboembolism prophylaxis, GDFT, lung protection mechanical ventilation strategies, transfusion guidelines, and continuous infusion of local anesthetic through the epidural catheter. Postoperative components included the following: continuous infusion of local anesthetics through epidural catheter, multimodal analgesia, early removal of nasogastric tube and Foley catheter, and early ambulation on postoperative day 1.

### Goal directed fluid therapy

**FIG. 1.**
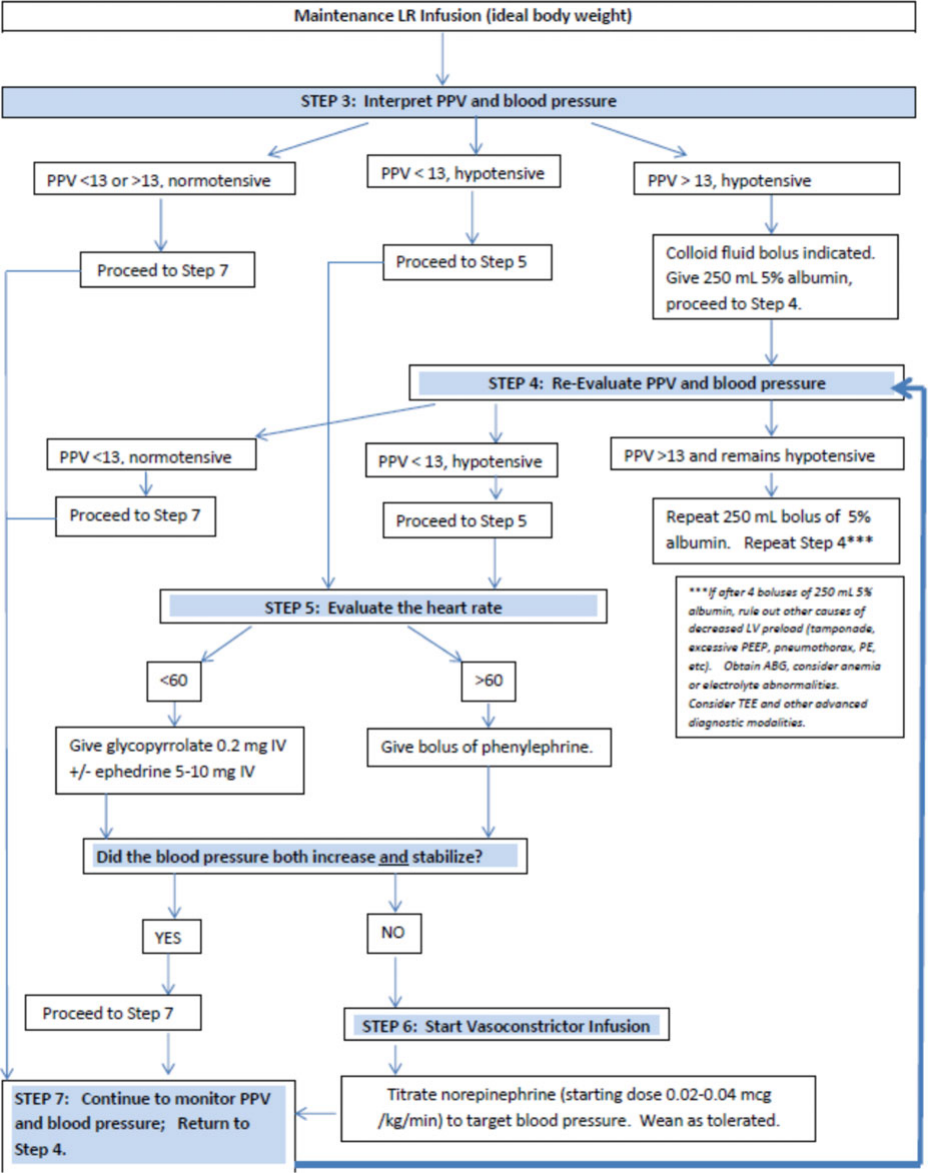
Goal directed fluid therapy algorithm in the pancreatic surgery enhanced recovery after surgery pathway. ABG, arterial blood gas; LR, lactated ringers; LV, left ventricle; PE, pulmonary embolism; PEEP, positive end expiratory pressure; PPV, pulse pressure variation; TEE, transesophageal electrocardiogram.

A key component of the ERAS pathway for pancreatic surgery was the application of a GDFT algorithm (Fig. 1) for management of intraoperative hypotension. Hypotension was defined as systolic blood pressure less than 20% of the patient's baseline.^23^ The first step in the GDFT algorithm was evaluation of preload using real-time interpretation of pulse pressure variation (PPV), as derived from arterial line waveform analysis (Philips^®^ IntelliVue MP70 monitor, Eindhoven, Netherlands).^24^ Limitations of accurate PPV interpretation include arrhythmia, small tidal volumes, and high positive end expiratory pressure (PEEP).^25^ A PPV of 13% or greater was interpreted as preload deficiency. If all other causes of preload deficiency were ruled out (i.e., excessive PEEP, RV failure, tension pneumothorax, large pulmonary embolism, vena cava compression, and so on), a fluid bolus was administered to treat hypovolemia and increase stroke volume. A fluid bolus of 5% albumin was used to avoid excess administration of salt, enhance intravascular volume through improved oncotic pressure, and limit edema formation. The increase in stroke volume subsequently increased cardiac output and ultimately increased systemic blood pressure. Hypotension in the setting of a PPV less than 9% implied normal preload and suggested another cause for hypotension such as low cardiac output (i.e., due to bradycardia) or low SVR. A PPV between 9% and 13% is a hemodynamic “gray zone,” and patient response to fluid administration in the setting of hypotension is less predictable.^26^ For the purposes of user simplicity, our GDFT used a binary decision regarding PPV: greater than 13% or less than 13%. The GDFT algorithm directed the provider to first evaluate heart rate; if the PPV was less than 13% and bradycardia was present, the recommended treatment was glycopyrrolate or ephedrine. If hypotension persisted in the setting of a PPV less than 13%, then the etiology was presumed to be low SVR. The algorithm then directed the provider to start a vasopressor infusion, specifically a norepinephrine infusion was most commonly used as first line, and vasopressin could be added if necessary.

### Statistical analysis

Patient demographics and intraoperative characteristics (including vasopressor usage) in the pre-ERAS and ERAS cohorts were compared using Fisher's exact and Wilcoxon-Mann-Whitney tests, where appropriate. A *p*-value <0.05 was considered significant. Log-binomial regression of the potential effect of ERAS on pancreatic leaks was performed to estimate the change in risk (i.e., risk ratios [RRs]). Pancreatic leaks were compared as presence versus absence, as well as clinically significant (classified as Grade B or C) versus clinically insignificant (Grade A) or no leak. The standard criteria to define leak grade, as validated by the International Study Group of Pancreatic Fistulas (ISGPF), were utilized.^27^

To account for potential confounding, log-binomial models were weighted by inverse probability of treatment weighting. Briefly, weights were estimated using multivariable logistic regression, which modeled the probability of treatment under ERAS using patient age, sex, procedure type, American Society of Anesthesiologists classification, body mass index, smoking status (categorized as current smoker, ex-smoker, and nonsmoker), diabetes mellitus, hypertension, chronic obstructive pulmonary disease (COPD), congestive heart failure (CHF), coronary artery disease (CAD), and chronic renal insufficiency. All continuous variables were modeled as restricted cubic splines. Weights were stabilized using the marginal (i.e., overall) probability of being treated under the ERAS pathway in the cohort. Similar methods were used to estimate the change in risk of leaks among patients who received vasopressors, compared to those who did not, regardless of the pathway in place; however, the weights were estimated using the covariates described above, as well as the amount of crystalloid fluids, colloid fluids, and units of blood given during surgery. Confidence intervals (CIs) were calculated using the robust variance sandwich estimators to account for the weighting. All analyses were performed using SAS (SAS, Inc., Cary, NC).

## Results

Of the 132 patients included in the study analysis, 74 (56.1%) were treated with ERAS pathway. There were no significant differences in patient demographics, comorbidities, or preoperative smoking status between the ERAS or pre-ERAS cohorts (Table 1). There was a significant increase in intraoperative vasopressor infusion usage among patients in the ERAS cohort (57% vs. 38%), *p* = 0.04. A significant increase in the median proportion of operative time on vasopressors in the ERAS and pre-ERAS cohorts was also seen, 27% versus 0%, *p* = 0.02. An increase in the amount of colloid fluids also increased after ERAS was implemented (median 1000 vs. 500 mL, *p* = 0.008). No other significant differences in pancreatic texture, pancreatic duct size, operative time, estimated blood loss, blood transfusions, and crystalloid administration were observed between the two cohorts (Table 2). Readmission rates and length of stay were both improved in the ERAS cohort 9.5% versus 15.5% and 8 days versus 10.2 days, respectively, but neither difference was statistically significant.

**Table 1.** **Patient Characteristics Before and After Implementation of an Enhanced Recovery After Surgery Pathway**

	Pre-ERAS 58 (44%)	Post-ERAS 74 (56%)	*p*-Value^a^
Age, in years, med (IQR)	60 (51–69)	63 (57–72)	0.11
Male, *n* (%)	27 (47)	43 (58)	0.22
Primary diagnosis, *n* (%)			
Adenocarcinoma^b^	32 (53)	38 (51)	0.73
Cystic lesion	11 (19)	18 (24)	0.53
Neuroendocrine tumor	7 (12)	10 (14)	0.99
Other^c^	8 (14)	8 (11)	0.60
BMI, kg/m^2^, med (IQR)	26 (23–32)	27 (25–31)	0.50
ASA, med (IQR)	3 (3–3)	3 (3–3)	0.39
Smoking status, *n* (%)			
Current	15 (26)	14 (19)	0.40
Former	16 (28)	35 (47)	**0.03**
Never	27 (47)	25 (34)	0.15
Comorbidities, *n* (%)			
Diabetes mellitus	24 (41)	23 (31)	0.20
Hypertension	29 (50)	49 (65)	0.11
COPD	5 (9)	6 (8)	0.99
CHF	1 (2)	1 (1)	0.99
CAD	3 (5)	11 (15)	0.09
Renal insufficiency	1 (2)	5 (7)	0.23

^a^ Fisher's exact and Wilcoxon-Mann-Whitney tests were performed, where appropriate; *p*-values <0.05 are denoted in bold.

^b^ Pancreatic or ampullary.

^c^ Includes adenoma/polyp, autoimmune pancreatitis, cholangiocarcinoma, and chronic pancreatitis.

ASA, American Society of Anesthesiologists classification; BMI, body mass index; CAD, coronary artery disease; CHF, congestive heart failure; COPD, chronic obstructive pulmonary disease; ERAS, enhanced recovery after surgery; IQR, interquartile range; med, median.

**Table 2.** **Intraoperative Characteristics Before and After Implementation of an Enhanced Recovery After Surgery Pathway**

	Pre-ERAS 58 (44%)	Post-ERAS 74 (56%)	*p*-Value^a^
Procedure, *n* (%)			
Whipple	41 (71)	46 (62)	0.36
Distal pancreatectomy	17 (29)	28 (38)	–
Pancreatic texture,^b^*n* (%)			
Hard	32 (80)	36 (78)	0.99
Soft	8 (20)	10 (22)	–
Pancreatic duct size,^b^ mm, med (IQR)	4 (3–4)	4 (3–4)	0.23
Estimated blood loss, mL, med (IQR)	500 (300–1000)	500 (200–700)	0.08
Units of blood products, *n* (%)			
0 U	42 (72)	62 (84)	0.14
≥1 U	16 (28)	12 (16)	–
Crystalloid fluids, mL, med (IQR)	3950 (2700–5200)	3920 (2550–5065)	0.72
Colloid fluids, mL, med (IQR)	500 (0–1000)	1000 (500–1250)	**0.0008**
Vasopressors used, *n* (%)	22 (38)	42 (57)	**0.04**
Vasopressor time,^c^ med (IQR)	0 (0–19)	27 (0–67)	**0.02**

^a^ Fisher's exact and Wilcoxon-Mann-Whitney tests were performed, where appropriate; *p*-values <0.05 are denoted in bold.

^b^ Measured in patients undergoing a Whipple only.

^c^ Percentage of OR time on vasopressors during surgery.

OR, operating room.

After stratifying by surgical procedure, increased vasopressor infusion usage was only observed in Whipple procedures (74% vs. 49%), *p* = 0.03. No change in vasopressor infusion usage was seen in distal pancreatectomies, 29% versus 12%, *p* = 0.28. Similarly, an increase in the median proportion of operative time on vasopressors was only observed among Whipple procedures (51% vs. 0%), *p* = 0.01. No increase in median proportion of operative time on vasopressors was observed among distal pancreatectomy procedures (0% vs. 0%), *p* = 0.17.

Overall incidence of pancreatic fistula diagnosed in a 6-month follow-up period for the combined pre-ERAS and ERAS cohorts was 15% (20/132 patients), 19 of which were considered clinically significant (Grade B or C). While patients undergoing Whipple who had soft gland texture were more likely to have a leak (22% vs. 9%), this difference was not statistically significant, *p* = 0.21. In addition, there was no difference in duct size between patients with and without leaks (median 3 vs. 4 mm, *p* = 0.14).

Compared to pre-ERAS implementation, no significant changes in the overall rate of leaks (RR 0.96, 95% CI 0.43–2.16, *p* = 0.92) or clinically significant leaks (RR 0.87, 95% CI 0.38–2.00, *p* = 0.74) were observed (Table 3). Even after inverse probability-of-treatment weighting, no changes in 6-month rate of leaks (RR 0.89, 95% CI 0.38–2.09, *p* = 0.79) or clinically significant leaks (RR 0.82, 95% CI 0.34–1.96, *p* = 0.65) were seen.

**Table 3.** **Six-Month Risk of Pancreatic Leak Following Pancreatic Surgery, Before and After Implementation of an Enhanced Recovery After Surgery Pathway**

	Risk of leak, %				
	Pre-ERAS	Post-ERAS	Risk ratio	95% CI^a^	*p*-Value
Any leak					
Crude	15.5	14.9	0.96	0.43–2.16	0.92
Weighted^b^	17.3	15.5	0.89	0.38–2.09	0.79
Significant leak					
Crude	15.5	13.5	0.87	0.38–2.00	0.74
Weighted^b^	17.3	14.1	0.82	0.34–1.96	0.65

^a^ CIs were calculated using the robust variance sandwich estimators.

^b^ Weighted for patient age, sex, procedure type, ASA classification, BMI, smoking status, diabetes, hypertension, COPD, CHF, CAD, and chronic renal insufficiency; all continuous variables were modeled as restricted cubic splines.

CI, confidence interval.

The direct effect of vasopressors on the 6-month rate of leaks, and significant leaks, was also assessed. In totum, 64 patients (48%) were treated with vasopressor infusion intraoperatively. The median proportion of operative time on vasopressors among all patients who received a vasopressor infusion was 62% (IQR 31–80%). Patients receiving vasopressors were significantly more likely to have a diagnosis of adenocarcinoma (61% vs. 22%, *p* < 0.0001). No other significant differences in patient or intraoperative characteristics were seen (data not shown). After weighting, there was still no evidence that using vasopressor infusions significantly increased the risk of leaks (RR 1.31, 95% CI 0.59–2.93, *p* = 0.51) or clinically significant leaks (RR 1.19, 95% CI 0.52–2.72, *p* = 0.68) after weighting (Table 4).

**Table 4.** **Six-Month Risk of Pancreatic Leak Among Patients Who Received Vasopressors During Pancreatic Surgery, Compared to Those Who Did Not**

	Risk of leak, %				
	Vasopressors	No vasopressors	Risk ratio	95% CI^a^	*p*-Value
Any leak					
Crude	17.2	13.2	1.30	0.58–2.93	0.53
Weighted^b^	16.0	21.0	1.31	0.59–2.93	0.51
Significant leak					
Crude	15.6	13.2	1.18	0.51–2.72	0.70
Weighted^b^	19.0	16.0	1.19	0.52–2.72	0.68

^a^ CIs were calculated using the robust variance sandwich estimators.

^b^ Weighted for patient age, sex, procedure type, ASA classification, BMI, smoking status, diabetes, hypertension, COPD, CHF, CAD, and chronic renal insufficiency; all continuous variables were modeled as restricted cubic splines.

## Discussion

The outcomes of this retrospective review of a prospectively initiated ERAS pancreatectomy protocol suggest that increased vasopressor use, as part of an ERAS pathway, does not increase the rate of pancreatic fistula. This challenges an established bias that vasopressor use increases the risk of anastomotic leak and pancreatic fistulas.

Appropriate healing of gastrointestinal anastomoses requires appropriate blood supply and oxygenation.^13–15^ Vasopressor is associated with splanchnic vasoconstriction and, thus, gastrointestinal hypoxia and injury.^16–18^ Vasopressors are often utilized in clinical situations such as shock, hypovolemia, sepsis, adrenal insufficiency, and other risk factors that predispose poor wound healing and anastomotic leaks.^28–31^ Controversy exists on whether vasopressors are associated with increased rates of anastomotic leaks. However, there is a paucity of evidence to support this theory. In addition, there is no evidence available in regard to pancreatic resection and pancreatic-intestinal anastomosis leaks in the setting of intraoperative vasopressor infusions. A retrospective review by Zakrison, et al. of 223 patients undergoing a variety of gastrointestinal anastomosis admitted to ICU after surgery revealed 22 (9.9%) patients with anastomotic leaks. Vasopressor use was associated with increased anastomotic leakage, *p* = 0.02. This difference was even more pronounced in patients requiring multiple vasopressors, *p* = 0.0008, and prolonged exposure, *p* = 0.0006, independent of APACHE II score. Multivariable analysis revealed that vasopressor exposure was associated with a leak with an odds ratio of 3.26 (95% CI 1.13–9.39).^19^ Limitations of this retro spective review included small sample size, only 22 leaks, and only 26 of the 223 patients exposed to vasopressors. Second, this retrospective review looked at prolonged postoperative vasopressor infusion, not intraoperative use.

Sultan, et al. published a retrospective review of 127 consecutive colorectal resections with anastomosis between 2000 and 2010 in a single Pakistani university hospital. Nineteen (15%) patients experienced a clinical leak. Bivariate analysis demonstrated that intraoperative use of vasopressor was associated with anastomotic leak, *p* = 0.04. On multivariable analysis, only smoking, left-sided resections, blood transfusions, and emergency surgeries remained independent risk factors for leak.^20^ The retrospective review was limited by the fact that 11% of these operations were emergent, and it is not clear if a disproportionate number of patients with intraoperative vasopressor use were emergent, which would place them at higher risk of leaks. Moreover, vasopressor use was no longer a risk factor on multivariable analysis, which suggests that there were confounding factors involved.

Fischer et al. retrospectively described trauma patients undergoing initial damage control laparotomies with colonic resection followed by colonic anastomosis in a staged manner. They reviewed 41 patients with anastomosis after damage control resection and found a 17% incidence of leaks. Fifty-seven percent of patients who had a leak required vasopressors at some point after damage control laparotomy and anastomosis, compared to only 12% of patients who did not have a leak, *p* = 0.02. None of the patients in either group had intraoperative vasopressor use.^21^ This is difficult to interpret with regard to the direct effects of vasopressors on leak, as none of the patients were on vasopressors during the anastomosis construction; thus, as the authors concluded, vasopressor use in this setting was simply a surrogate of ongoing shock.

Adanir, et al. studied the role of vasopressors on anastomotic leaks in an animal model. Forty-two male New Zealand rabbits had ileocolic anastomoses and were either given no vasopressors or an experimental dose of 5, 10, 15, 20, or 25 μg/kg/h dopamine infusions for 2 h. On postoperative day 4, relaparotomy and *in vivo* burst pressure analysis followed by resection and analysis of hydroxyproline and collagen tissue levels (used as surrogates of appropriate healing) in the anastomoses were compared. No changes to collagen or hydroxyproline levels were noted between the groups, and no decrease in bursting pressures noted. Surprisingly, at the high doses of dopamine (>20 μg/kg), the anastomosis exerted higher bursting pressures. This demonstrates that in the absence of hypovolemia, vasopressor use did not increase tendency toward anastomotic leak.^22^

The GDFT algorithm, as part of an ERAS pathway, includes the use of vasopressors when clinically appropriate (i.e., once the patient is considered to not be preload deficient). As a GDFT algorithm was not used in the pre-ERAS group, these patients received fewer vasopressor infusions as part of their hemodynamic management. Patients in the ERAS cohort spent a longer duration on vasopressor infusions than patients in the pre-ERAS cohort. One limitation of our study is that we did not evaluate the effect of intermittent vasopressor boluses that were given intraoperatively. This was done for two reasons. The first is that two different vasopressors (phenylephrine and vasopressin) were commonly used intraoperatively for intermittent bolus. This limits the ability to compare total dosages administered across vasopressors. The second reason intermittent boluses were not included is that the impact of this practice is likely minimal on the development of pancreatic fistula incidence. It is reasonable to assume that the negative impact of vasopressors on end organ perfusion is related to duration of exposure to vasopressor, especially in patients who are under-resuscitated. We observed that the use of intraoperative vasopressor infusions, when used in patients who are assessed to be euvolemic by the GDFT algorithm, does not significantly increase the risk of pancreatic fistulas (RR 1.31, *p* = 0.51).

We have successfully demonstrated that there was no increase in the 6-month risk of pancreatic fistula between the ERAS and pre-ERAS cohorts. Overall, the risk of fistula was slightly lower 11/74 (15%) in the ERAS cohort versus 9/58 (16%) in the pre-ERAS cohort, but this was not statistically significant. As GDFT was not the only change in patient management after implementation of an ERAS pathway for pancreatectomy, it is conceivable that other modifications counteracted the negative effects of vasopressors and thus we could not identify a difference in fistula incidence between groups. To account for that possibility, we directly evaluated the effects of vasopressor use on fistula risk, independent of ERAS status. These analyses demonstrated that patients who received intraoperative vasopressor infusion had no significant difference in the risk of pancreatic fistula compared to those who did not receive vasopressor infusions. This suggests that the lack of difference is not related to other effects exerted by the implementation of the ERAS protocol.

Another limitation is our low event rate with only 20 patients experiencing pancreatic fistulas, which limits our ability to detect small, but potentially clinically relevant, differences. Furthermore, our study combines both distal pancreatectomies and pancreaticoduodenectomy leak rates, and this limits the conclusions that can be drawn about either procedure individually. Certainly, individual comparisons of these procedures would be more consistent. Finally, our study did not accurately capture grade A ISGPF leaks, as it is not our routine practice to measure drain amylase on postoperative day number 3. All the analyses were done for both all leaks and clinically significant (grade B and C) leaks, with similar results, but our conclusions should stress that our study was designed to only delineate clinically significant leaks.

Our rates of pancreatic fistula and lack of difference between the cohorts are both consistent with previous reports of pancreatic ERAS protocols. Coolsen et al. published a meta-analysis of ERAS protocols for pancreaticoduodenectomies.^32^ The analysis demonstrated shorter length of stay and decreased complication rate in the ERAS cohorts, and in regard to pancreatic fistula, rates ranged from 7% to 25% with no statistically significant differences. Likewise, other meta-analysis of ERAS anastomotic leaks failed to show a difference between ERAS and control cohorts in both obesity and colorectal surgeries.^33,34^

## Conclusions

The negative effects of vasopressors on anastomotic complications and pancreatic fistulas have been long suspected. Still, little data exist on the actual impact vasopressors have on anastomotic leaks and even less on rates of pancreatic fistulas. We have shown that increased use of intraoperative vasopressor infusions, as part of an ERAS pathway for pancreatic surgery, does not increase the risk of pancreatic fistulas.

## Abbreviations Used

BMI: body mass index
CAD: coronary artery disease
CHF: congestive heart failure
CI: confidence interval
COPD: chronic obstructive pulmonary disease
ERAS: enhanced recovery after surgery
GDFT: goal directed fluid therapy
IQR: interquartile range
ISGPF: International Study Group of Pancreatic Fistulas
PEEP: positive end expiratory pressure
RR: risk ratio
SVR: systemic vascular resistance
